# The AMPylase FIC-1 modulates TGF-β signaling in *Caenorhabditis elegans*

**DOI:** 10.3389/fnmol.2022.912734

**Published:** 2022-11-24

**Authors:** Mirella A. Hernandez-Lima, Margaret Champion, Zachary Mattiola, Matthias C. Truttmann

**Affiliations:** ^1^Neuroscience Graduate Program, University of Michigan, Ann Arbor, MI, United States; ^2^Department of Molecular and Integrative Physiology, University of Michigan, Ann Arbor, MI, United States; ^3^Geriatrics Center, University of Michigan, Ann Arbor, MI, United States

**Keywords:** AMPylation/adenylylation, chaperones, proteostasis, transforming growth factor, FIC activity

## Abstract

Post-translational protein modifications are essential for the spatio-temporal regulation of protein function. In this study, we examine how the activity of the *Caenorhabditis elegans* AMPylase FIC-1 modulates physiological processes *in vivo*. We find that over-expression (OE) of the constitutive AMPylase FIC-1(E274G) impairs *C. elegans* development, fertility, and stress resilience. We also show that FIC-1(E274G) OE inhibits pathogen avoidance behavior by selectively suppressing production of the Transforming Growth Factor-β (TGF-β) ligands DAF-7 and DBL-1 in ASI sensory neurons. Finally, we demonstrate that FIC-1 contributes to the regulation of adult body growth, cholinergic neuron function, and larval entry into dauer stage; all processes controlled by TGF-β signaling. Together, our results suggest a role for FIC-1 in regulating TGF-β signaling in *C. elegans*.

## Introduction

Post-translational protein modifications (PTMs) are fundamental components of the proteome that enhance functional diversity and play critical roles in orchestrating cellular processes. Alterations in the cellular PTM machinery are implicated in many major diseases including neurodegenerative diseases and cancer (Berson et al., 2018; Ferguson and Gray, 2018; MacDonald, 2018). Epigenetic imprinting of parental histones expands the impact of PTMs to trans-generational signaling (Hanna and Kelsey, 2017), which provides organisms with an effective mechanism to inherit regulatory traits that might offer their offspring selective advantages or disadvantages.

The human AMPylase, FICD, and its *C. elegans* ortholog, FIC-1, belong to a family of fic domain-containing AMPylases present as a single copy in metazoans (Truttmann and Ploegh, 2017; Casey and Orth, 2018; Chatterjee and Truttmann, 2021). Fic AMPylases are bi-functional, catalyzing both the addition (AMPylation) and the removal (deAMPylation) of an adenosine monophosphate (AMP) moiety to serine or threonine hydroxyl groups of target proteins using a single active site (Ham et al., 2014; Preissler et al., 2015, 2017a,b; Sanyal et al., 2015). The switch between AMPylation and deAMPylation is triggered by enzyme dimerization, exchange of Mg^2+^ for Ca^2+^ ions in the active site, and a conformational switch (Casey et al., 2018; Casey and Orth, 2018; Perera et al., 2019; Fauser et al., 2021; Perera et al., 2021). Replacement of an inhibitory glutamate residue with a glycine residue (FICD(E234G), FIC-1(E274G)) renders the enzymes into constitutively active AMPylases (Engel et al., 2012; Truttmann et al., 2016).The precise cellular conditions and mechanism regulating the switch between AMPylation and deAMPylation remain poorly understood. However, *in vitro* studies have shown that the treatment of human cells with the protein synthesis inhibitor cycloheximide (CHX) increases, while acute ER stress decreases protein AMPylation, suggesting that fic AMPylases are involved in proteostasis regulation.

FICD AMPylates and deAMPylates the ER-resident HSP70 family chaperone BiP on Thr518 and Thr356, which serves as a mechanism to regulate client binding and refolding activity of the cellular BiP pool (Truttmann and Ploegh, 2017; Casey and Orth, 2018; Chatterjee and Truttmann, 2021). Similarly, FIC-1 AMPylates the two presumable functionally redundant *C. elegans* BiP orthologs HSP-3 and HSP-4 in the ER. In addition to BiP, an increasing number of additional fic AMPylase targets have recently been described, including other HSP70 family chaperones, translation elongation factor Eef-1A, and cathepsin family proteases (Lewallen et al., 2014; Broncel et al., 2015; Truttmann et al., 2016, 2017; Gulen et al., 2020; Kielkowski et al., 2020a,b; Nitika et al., 2020).

Previous studies have linked the loss of fic AMPylase activity to defects in neurotransmitter recycling (Rahman et al., 2012), eyesight in *Drosophila* (Rahman et al., 2012; Moehlman et al., 2018), ER stress resilience (Ham et al., 2014; Preissler et al., 2015; Sanyal et al., 2015), neuronal differentiation (Kielkowski et al., 2020b), and cytokine secretion (McCaul et al., 2021). Work by several groups also established that excessive AMPylation caused by the over-expression of a constitutive fic AMPylase is lethal for human (Bunney et al., 2014; Sanyal et al., 2015; Truttmann et al., 2015) and *S. cerevisiae* cells (Truttmann et al., 2017), flies (Casey et al., 2018), and *C. elegans* embryos (Truttmann et al., 2018). Cell death does not occur as a consequence of cellular ATP pool depletion due to increased AMPylase activity but rather appears to involve apoptotic signaling cascades (Sanyal et al., 2015). However, our understanding of the broader physiological and mechanistic implications of excessive AMPylation remain limited.

Here, we examine the physiological consequences of FIC-1(E274G) over-expression in *C. elegans* and implicate it in the regulation of reproduction, pathogen avoidance, and body size regulation. FIC-1(E274G) OE directly alters pathogen avoidance behavior by inhibiting the production of TGF-β ligands DBL-1 and DAF-7 in ASI sensory neurons. RNAi-mediated knock-down (kd) of the major cytosolic HSP70 family chaperone, HSP-1, phenocopies the consequences of FIC-1(E274G) OE on pathogen avoidance. Finally, we confirm a role for FIC-1-mediated regulation of TGF-β signaling in pathogen avoidance and dauer entry processes under physiological conditions. Our work highlights a contribution of FIC-1 activity and HSP70 family chaperones in modulating TGF-β signaling *in vivo*.

## Results

### FIC-1(E274G)::HA OE inhibits *Caenorhabditis elegans* development and reproduction

**Figure 1:**
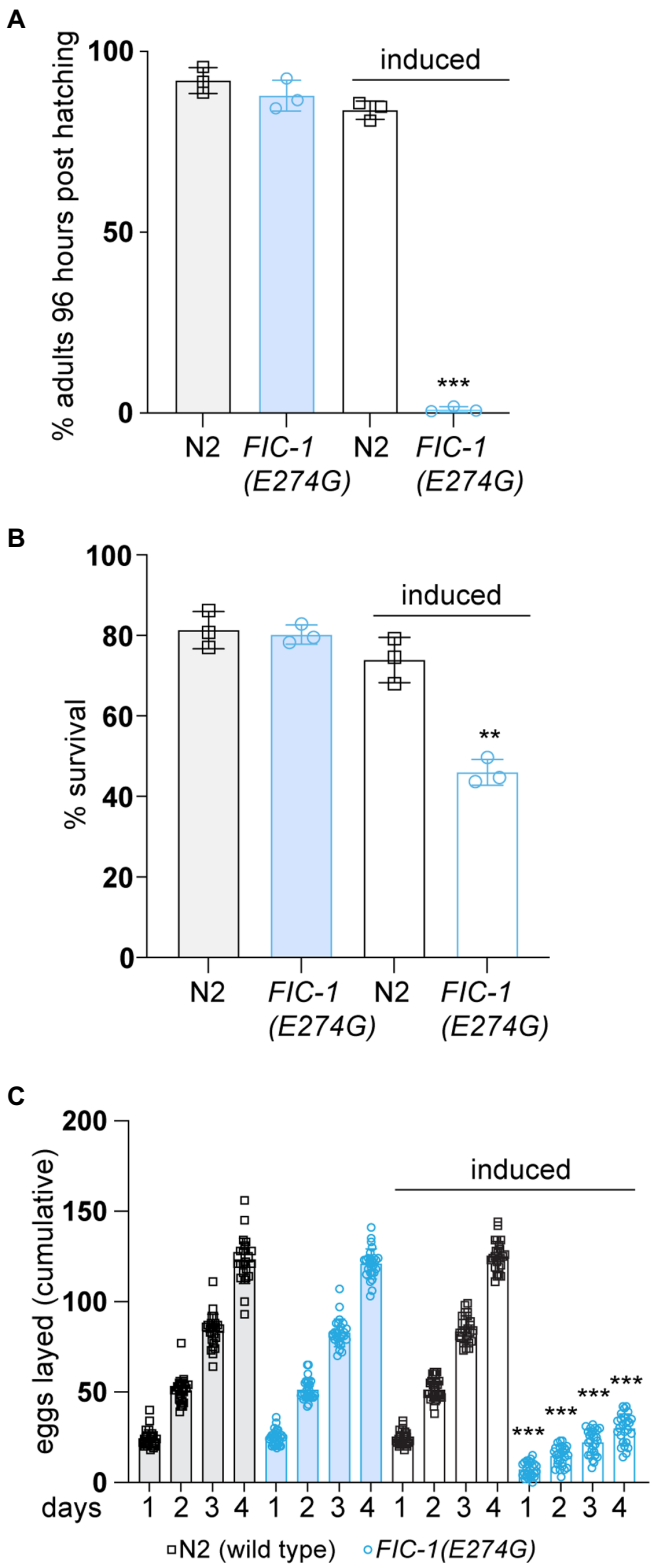
FIC-1(E274G)::HA OE impairs development, stress tolerance, and reproduction. **(A)** Induced and non-induced N2 and *nIs774* eggs were placed on NGM culture plates and incubated at 20°C. After 96 h, number of adults was determined and compared to initial number of eggs on each plate (*n* > 100/plate). **(B)** L4 animals were incubated at 30°C for 2 h. Following 30 min of recovery, worm survival was measured. **(C)** Egg laying of individual animals during the first 96 h of adulthood. Individually-kept animals (3 replicates, 9 animals per replicate/genotype) were transferred to fresh NGM growth plates in 24 h intervals and number of eggs was quantified by microscopy. For **(A,B)**: Each datapoint represents a biological replicate with >100 animals per replicate and condition. Error bars represent SD. Statistical significance (*p*-values) were calculated using unpaired *t*-tests, comparing induced N2 with induced nIs774 groups. **p < 0.01, ***p < 0.001, not significant (ns) *p* > 0.05.

We and others previously observed that over-expression (OE) of the constitutive-active fic AMPylase impairs stress signaling and leads to cell death in *S. cerevisiae*, *C. elegans*, and cultured human cells (Bunney et al., 2014; Sanyal et al., 2015; Truttmann et al., 2015, 2017). In *S. cerevisiae* and human cells, AMPylase OE resulted in the activation of a strong heat-shock response, which was accompanied by a decline in proteostasis. The goal of this study was to better understand the physiological consequences of FIC-1(E274G) OE on *C. elegans* physiology. We used a *C. elegans* strain carrying the allele *nIs774*, which allows for the inducible and ubiquitous expression of the constitutive AMPylase mutant FIC-1(E274G) with a C-terminal HA-tag (FIC-1(E274G)::HA) in *C. elegans*. Control experiments showed that FIC-1(E274G)::HA is efficiently expressed from *nIs774* as soon as 2 h after induction (Supplementary Figure S1A). Using *nIs774* and N2 wild type animals, we first performed development assays. In these experiments, we inducibly expressed FIC-1(E274G)::HA in synchronized *C. elegans* embryos and followed larval development until adulthood. We found that FIC-1(E274G)::HA OE completely halted larval development beyond the L1 stage even after 96 h at 20°C (Figure 1A). In contrast, uninduced control and induced wild-type embryos developed into fertile adults within 72 h at 20°C. Next, we tested how FIC-1(E274G)::HA OE affects stress tolerance. We incubated uninduced and induced wild type and *nIs774* animals at 30°C for 2 h, a temperature previously shown to induce the heat-shock response and heat-associate stress in *C. elegans* (Truttmann et al., 2017). Following 30 min of recovery at 20°C, we observed a significant reduction in the survival of FIC-1(E274G)::HA OE animals compared to uninduced and wild type controls (Figure 1B). Finally, we assessed whether FIC-1(E274G)::HA OE alters *C. elegans* reproduction. For this, we first induced FIC-1(E274G)::HA OE in day 1 adults and quantified the number of eggs laid per worm during the following 4 days. Our analysis showed that FIC-1(E274G)::HA OE significantly reduced both the number of eggs laid each day and the total number of eggs laid within 96 h post-induction (Figure 1C; Supplementary Table S1B). Collectively, our results show that FIC-1(E274G)::HA OE interferes with *C. elegans* development, stress resistance, and reproduction.

### Fic-1(E274G)::Ha OE inhibits pathogen avoidance behavior in *Caenorhabditis elegans*

**Figure 2:**
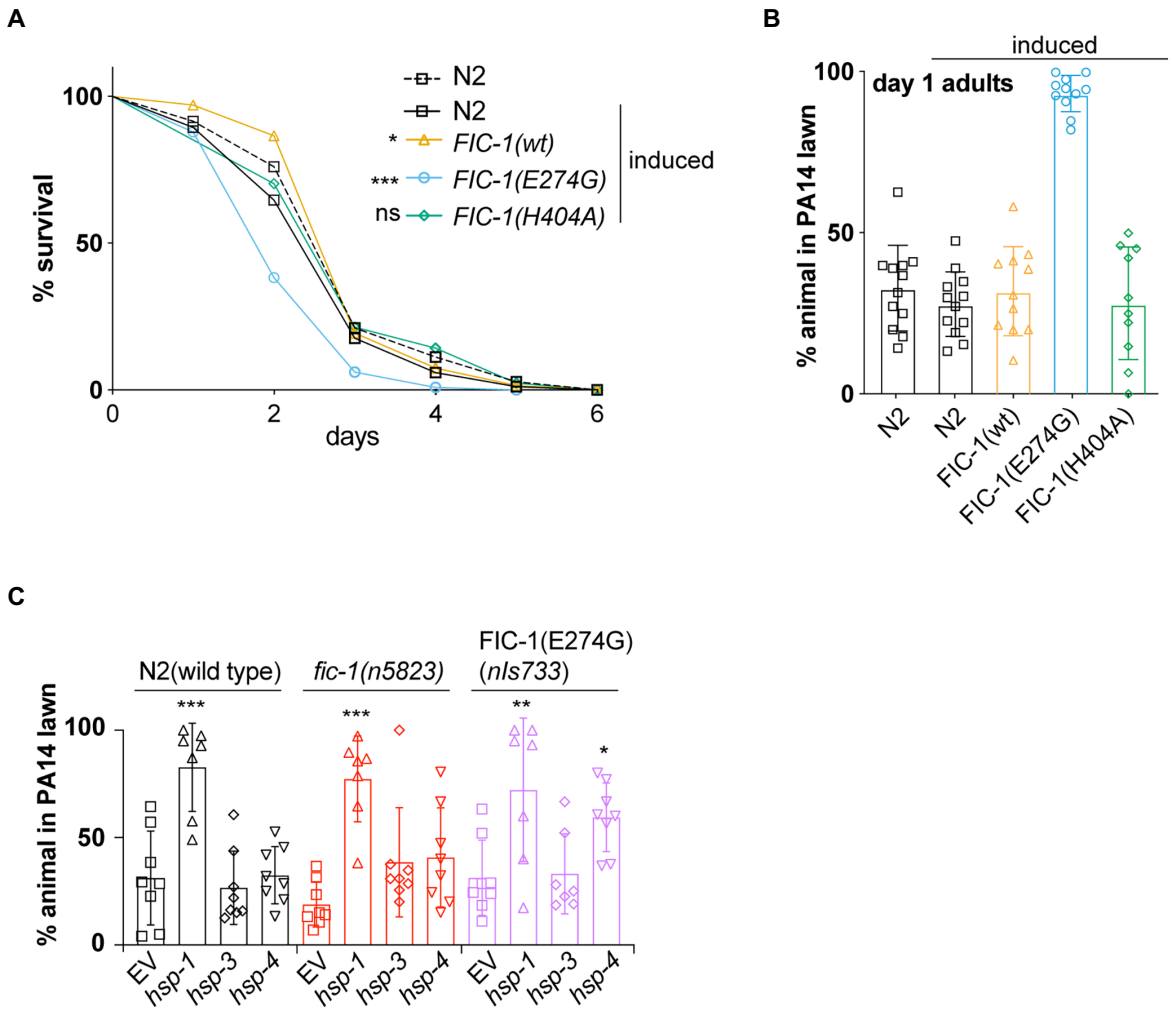
FIC-1(E274G)::HA OE inhibits pathogen avoidance behavior in *Caenorhabditis elegans*. **(A)** Survival of indicated strains on *Pseudomonas aeruginosa* PA14 plates. *n* > 80 animals per condition. Statistical significance was calculated using Gehan-Breslow-Wilcoxon test. **(B)** Synchronized young adults of indicated strains were placed in the center of a *P. aeruginosa* PA 14 lawn and incubated for 20 h. Number of worms remaining and leaving *P. aeruginosa PA 14* lawn was determined. **(C)** Synchronized young adults of indicated strains were treated with indicated RNAis for 48 h and subsequently placed in the center of a *P. aeruginosa* PA 14 lawn and incubated for 20 h. For **(B,C)**: Each datapoint represents a biological replicate with >20 animals per replicate and condition. Statistical significance was calculated using Kaplan–Meier test **(A)**, unpaired *t*-tests comparing induced N2 with induced *nIs774* groups **(B)** and ANOVA test with multiple comparisons, using wild type as reference **(C)**. *p < 0.05, **p < 0.01, ***p < 0.001, not significant (ns) *p* > 0.05.

In previous work, we showed that endogenous-level expression of FIC-1(E274G) in *C. elegans* modulates innate immune responses to the bacterial pathogen *Pseudomonas aeruginosa* isolate PA14 (Truttmann et al., 2016). We now tested how FIC-1(E274G)::HA OE affects *C. elegans* tolerance to *P. aeruginosa* PA14 in lifespan assays. We predicted that N2 wild type and *nIs774* worms released in the center of *P. aeruginosa* PA14 lawns would exhibit a protective behavioral avoidance response to *P. aeruginosa* PA14 but succumb to the infection within ~4 days. However, we found that OE of FIC-1(E274G)::HA, but not wild-type FIC-1 (*nIs776*) or catalytically inactive FIC-1(H404A) (*nIs778*) significantly reduced *C. elegans* survival on *P. aeruginosa* PA14 (Figure 2A), despite being expressed at similar or higher levels than FIC-1(E274G) (Supplementary Figure S2B). We also observed a dose-dependent decline in *nIs774* animal survival on *P. aeruginosa* PA14, with the decrease in survival correlating with increasing FIC-1(E274G)::HA expression (Supplementary Figure S2A). Interestingly, FIC-1(E274G)::HA OE significantly impaired the behavioral avoidance response in both larval (Supplementary Figure S2D) and adult (Figure 2B; Supplementary Figure S2C) animals. We also confirmed that FIC-1(E274G)::HA OE did not reduce *nIs774* worm motility (Supplementary Figure S2E), thus excluding motility defects as a reason for the failure to induce aversive behavior.

We previously showed that FIC-1(E274G) preferentially modifies and thus interferes with the function of HSP70 family chaperones HSP-3, and HSP-4 (both ER-resident) and HSP-1 (cytosolic) *in vitro*. We thus tested whether RNAi-mediated knock-down (kd) of *hsp-1*, *hsp-3*, and *hsp-4* mimics the consequences of FIC-1(E274G)::HA OE. We grew N2 wild type, *fic-1* null (*n5823*) and *fic-1*p::FIC-1(E274G) (*nIs733*) animals, in which FIC-1(E274G) is expressed at physiological levels, on *E. coli* HT115 cells expressing dsRNA precursors specific for *hsp-1*, *hsp-3*, and *hsp-4,* respectively. Following dsRNA uptake by feeding, these molecules are processed by the worm’s DICER complex, generating small interfering RNA (siRNA) molecules that induce knock down (kd) of target gene expression (Conte et al., 2015). After 24 h, we transferred the animals to *P. aeruginosa* PA14 lawns and analyzed how chaperone kd alters aversive behavior. We found that in all genetic backgrounds, kd of *hsp-1*, but not *hsp-3* abolished the behavioral avoidance response to *P. aeruginosa* PA14 (Figure 2C). Interestingly, kd of *hsp-4* in a *nIs733,* but not a wild type or *n5823* background also significantly impaired the induction of aversive behavior. Taken as a whole, these findings suggest a novel link between FIC-1-mediated AMPylation of HSP-3/HSP-4 and the regulation of behavioral responses in *C. elegans*.

### Fic-1(E274G)::Ha OE prevents DAF-7 and DBL-1, but not DAF-28 expression in ASJ neurons

**Figure 3:**
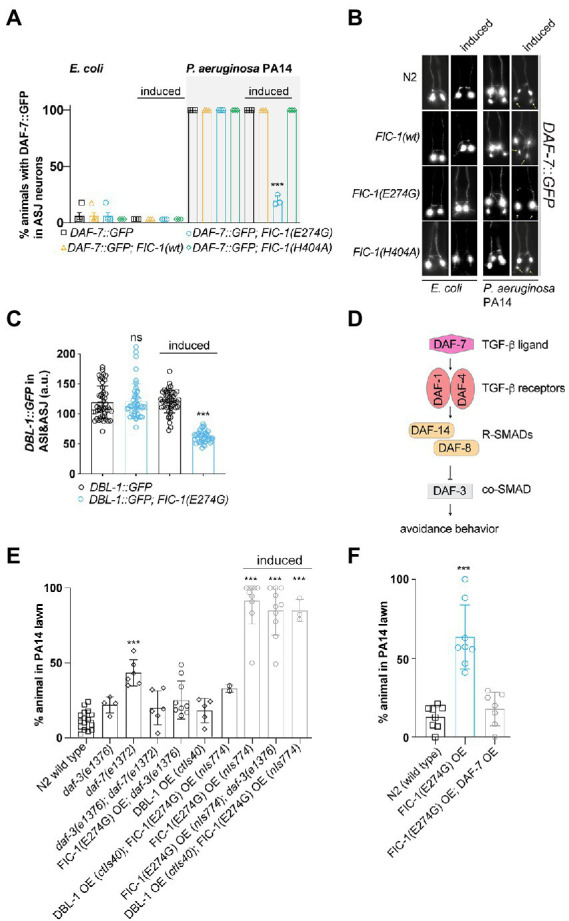
FIC-1(E274G)::HA OE suppresses TGF-β signaling in ASJ neurons in response to *P. aeruginosa* PA14 exposure. **(A,B)** Induced and non-induced strains were placed on *E. coli* OP50 or *P. aeuruginosa* PA14 lawns and incubated for 15 h, after which we determined the number of GFP-expressing in ASJ neurons. Each datapoint represents a biological replicate with >10 animals per replicate and condition. **(B)** shows representative images. Yellow asterisks point at ASJ neurons. Scale bar = 30 μm. Error bars represent SD. Statistical significance was calculated using unpaired *t*-test, comparing *ksIs2* to *ksIs2*;*nIs774*. **(C)** Induced and non-induced strains were placed on *P. aeuruginosa* PA14 lawns and incubated for 15 h, after which we determined the number of DBL-1::GFP-expressing ASI and ASJ neurons (*n* > 30 for each condition). Each datapoint in **(C)** represents a single animal measurement. Data was acquired in three biological replicates with >10 animals per replicate. **(D)** Canonical DAF-7/TGF-β and DBL-1/TGF-β signaling pathways in *C. elegans*. **(E,F)** Synchronized young adults of indicated strains were transferred to assay plates containing a *P. aeruginosa* PA 14 lawn and incubated for 20 h. Error bars represent SD. Each datapoint represents a biological replicate with >20 animals per replicate and condition. Statistical significance was calculated using unpaired *t*-test, comparing *ksIs2* to *ksIs2*;*nIs774* **(A)**, Mann–Whitney test comparing *ctIs40* to *ctIs40*; *nIs774* **(C)***, and* one-way ANOVA test with multiple comparisons using wild type as reference **(D)**. ***p < 0.001, not significant (ns) *p* > 0.05.

In *C. elegans*, exposure to *P. aeruginosa* PA14 induces the expression of the TGF-β ligand DAF-7 in ASI neurons, which signals to RIM/RIC interneurons to promote protective avoidance behavior (Meisel et al., 2014; Park et al., 2020). Based on our results, we hypothesized that FIC-1(E274G)::HA OE dysregulates TGF-β signaling processes in ASJ neurons in response to *P. aeruginosa* PA14 exposure. To explore this possibility, we crossed alleles *nIs774*, *nIs776*, and *nIs778* into a strain containing a *daf-7*p::GFP reporter (*ksIs2*) and quantified GFP expression in chemosensory ASI and ASJ neurons. When grown on *E. coli* OP50 lawns, GFP expression from the *daf-7*p:gfp reporter was only detected in ASI neurons, confirming previous findings (Meisel et al., 2014; Figures 3A,B). In contrast, animals exposed to *P. aeruginosa* PA14 showed a robust induction of the *daf-7*p::GFP reporter in ASJ neurons within 24 h. Strikingly, this induction was almost completely suppressed in FIC-1(E274G)::HA OE animals (Figures 3A,B). To exclude that these results were due to a global downregulation of protein translation in ASJ, we analyzed how FIC-1(E2774G)::HA OE alters expression of the DAF-28::GFP expression reporter from allele *svIs69*. DAF-28 is an insulin-like protein that is expressed in ASI and ASJ neurons and contributes to DAF-2-dependent signaling cascades (Li et al., 2003). Our results showed that, indeed, FIC-1(E2774G)::HA OE did not alter DAF-28::GFP expression in ASI or ASJ neurons (Supplementary Figure S3A). Next, we determined whether FIC-1(E274G)::HA OE interferes with the expression of DBL-1, a second major TGF-β ligand regulating gene expression in response to numerous bacterial pathogens, including *E. coli*, *E. faecalis*, and *P. aeruginosa* PA14 (Meisel et al., 2014; Park et al., 2020). For this, we crossed *nIs774* into animals expressing DBL-1::GFP from the allele *ctIs43* and quantified DBL-1::GFP signal in ASI and ASJ neurons. We found that FIC-1(E2774G)::HA OE significantly decreased DBL-1::GFP levels in both ASI and ASJ neurons (Figure 3C; Supplementary Figure S3B). We also observed that animals expressing DBL-1::GFP showed reduced tolerance to FIC-1(E274G)::HA OE, resulting in an almost complete loss of these populations 3 days following induction of FIC-1(E274G)::HA (Supplementary Figure S3C). Additional *P. aeruginosa* PA14 avoidance assays testing worms with knock-downs of *daf-1*, *daf-2*, *daf-4*, *sma-3*, and *sma-4* further supported our conclusion that FIC-1(E274G)::HA OE alters pathogen avoidance by engaging DAF-7-depenent TGF-β signaling (Supplementary Figure S3E).

Previous work by Meisler et al. established that the TGF-β type I receptor DAF-1, the R-SMAD DAF-8, and the co-SMAD DAF-3 are critical for mediating the physiological consequences of *P. aeruginosa* PA14-induced DAF-7 expression in ASJ neurons (Meisel et al., 2014; Figure 3D). They found that *daf-1* and *daf-8* mutants also displayed avoidance defects when exposed to *P. aeruginosa* PA14, whereas *daf-3* deficiency was sufficient to suppress *daf-7* null-mediated loss of avoidance. In avoidance assays, we confirmed that *daf-7*(*e1372*) animals showed impairments in protective aversive behavior, which was significantly improved in *daf-3*(*e1376*);*daf-7*(*e1372*) double mutants (Figure 3E). FIC-1(E274G)::HA OE in a *daf-3*(*e1376*) background did not significantly improve pathogen avoidance. Interestingly, OE of the TGF-β ligand DAF-7 (Figure 3F), but not DBL-1 was sufficient to rescue the loss of aversive behavior toward *P. aeruginosa* PA14 under FIC-1(E274G)::HA OE conditions (Figure 3E). However, DAF-7 OE was not sufficient to prevent FIC-1(E274G)::HA OE-dependent development impairments (Supplementary Figure S3D), suggesting that additional pathways critical for larval growth and maturation may be impacted by FIC-1(E274G)::HA OE. Taken together, these findings suggest that FIC-1(E274G)::HA OE interferes with the expression of TGF-β family ligands in ASI and ASJ neurons, which explains, in part, the observed loss of aversive behavior in response to *P. aeruginosa* PA14 exposure.

### Fic-1(E274G)::Ha OE induces small body phenotype, interferes with sensory perception in cholinergic neurons, and promotes dauer entry

**Figure 4:**
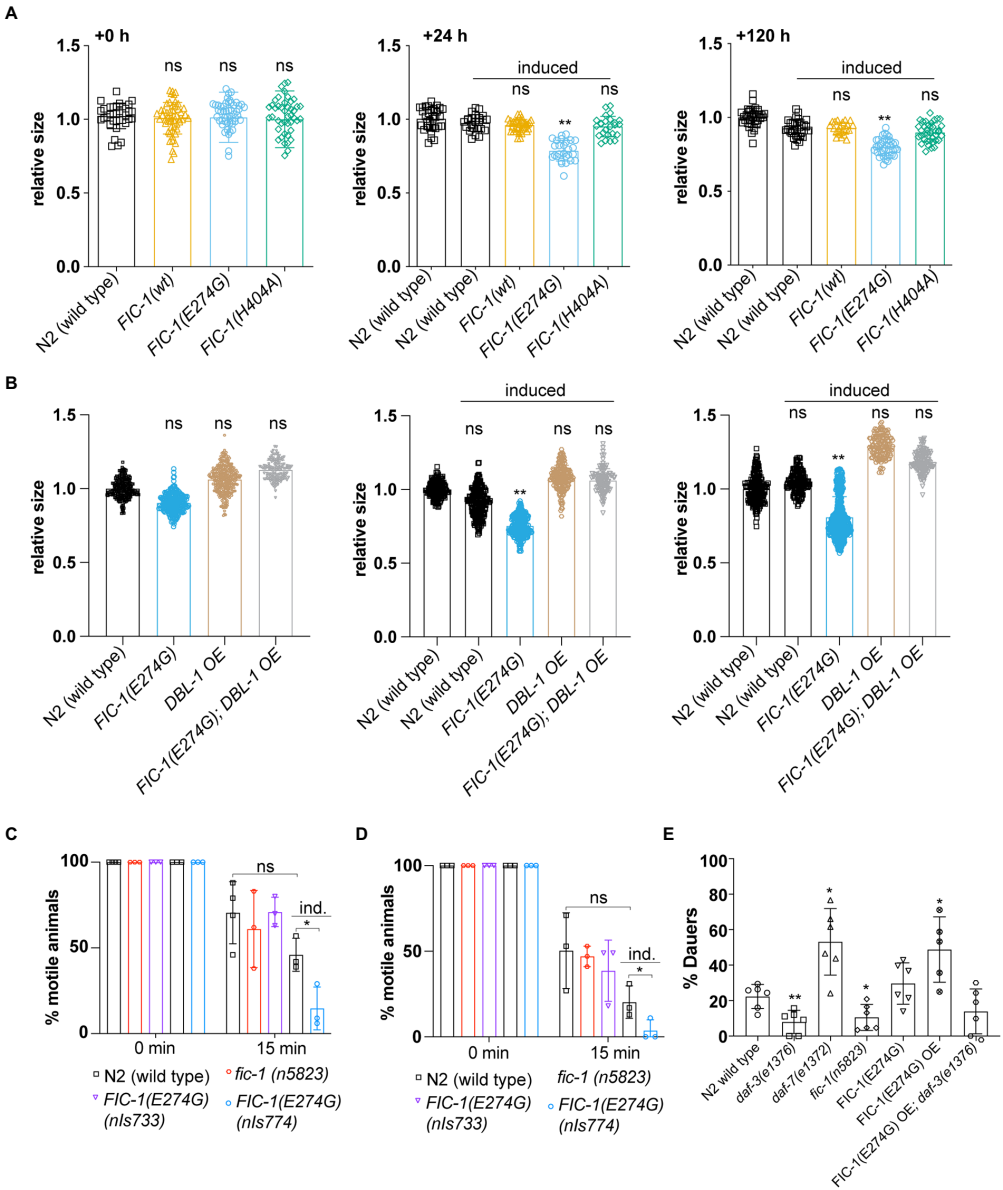
FIC-1(E274G)::HA OE interferes with TGF-β signaling-dependent physiological events. **(A,B)** Relative worm size at indicated timepoints post induction of transgene expression. Worm size was measured using Fiji. Each datapoint represents a single animal. Data was aquired in three biological replicates with >10 animals per replicate. **(C,D)** Indicated strains were incubated for 15 min in 0.5% (v/v) Nicotine **(C)** or 2 mM Tetramisole **(D)**, after which we determined the number of worms that were not paralyzed. Ind: induced **(E)** Indicated strains were transferred to 27°C as L1 larvae to follow dauer entry. For **(C–E)**: Each datapoint represents a biological replicate with >20 animals per replicate and condition. Error bars represent SD. Statistical significance was calculated using unpaired *t*-tests. **p < 0.01, *p < 0.05, not significant (ns) *p* > 0.05.

DBL-1 signaling is essential for worm body size control: While DBL-1 deficiency causes small body size, DBL-1 over-expression leads to increased body size (Morita et al., 2002). Since we observed that FIC-1(E274G)::HA OE suppressed DBL-1::GFP production in ASI and ASJ neurons, we hypothesized that the same condition should result in a decrease in worm body length. To test this prediction, we measured the major body axis of control worms and animals over-expressing FIC-1(E274G):HA (*nIs774*), FIC-1 wild type (*nIs776*) or FIC-1(H404A) (*nIs778*) 24 and 120 h after OE induction on day 1 of adulthood. We found that, as expected, FIC-1(E274G)::HA OE significantly reduced worm growth already within the first 24 h after OE induction, with a significant difference in body size persisting over a period of 120 h (Figure 4A). Notably, over-expression of DBL-1 was sufficient to prevent the FIC-1(E274G)::HA OE-promoted small (sma) phenotype (Figure 4B). Next, we tested whether FIC-1(E274G)::HA OE interferes with sensory perception in cholinergic neurons, as these neurons secrete DBL-1 to excite nearby body muscles. Null mutations in DBL-1 signaling components are hypersensitive to cholinergic agonists, including the anthelmintics Tetramisole and nicotine (Lewis et al., 1980). In a *C. elegans* liquid culture setup, we exposed uninduced and induced control and *nIs774* animals to 0.5% (v/v) Nicotine (Figure 4C), and 2 mM Tetramisole (Figure 4D). We found that FIC-1(E274G)::HA OE significantly reduced motility of animals exposed to either molecule as early as 15 min after experimental start. We also confirmed that these results were not due to defects of FIC-1(E274G)::HA expressing animals in cilia structures by analyzing cilia architecture and integrity in dye loading assays (Supplementary Figure S4A). Finally, we examined whether FIC-1(E274G)::HA OE promotes dauer entry when induced in L1 larvae. Because *daf-7* mutant animals show a significant increase in entering the dauer stage when cultured at temperatures above 25°C, we predicted that FIC-1(E274G)::HA OE would lead to increased dauer larvae formation as well. In our experiments, we quantified dauer entry at 20°C (Supplementary Figure S4B) and 27°C (Figure 4E) and found that FIC-1(E274G)::HA OE L1 larvae were significantly more likely to enter the dauer stage than control L1 larvae. Importantly, FIC-1(E274G)::HA OE in a dauer-deficient *daf-3*(*e1376*) background resulted in the inhibition of dauer formation. This confirms that FIC-1(E274G)::HA OE-triggered dauer entry signaling through the canonical *daf-7*-dependent TGF-β pathway. We also observed that *fic-1* deficiency significantly decreased dauer larvae formation, providing strong support for a physiological role for *fic-1* in regulating TGF-β signaling. Taken as a whole, our results show that FIC-1(E274G)::HA OE has a profound effect on DBL-1-and DAF-7-dependent TGF-β signaling that goes beyond aspects of innate immunity regulation.

### FIC-1(E274G)::HA OE changes expression of TGF-β signaling pathway members

**Figure 5:**
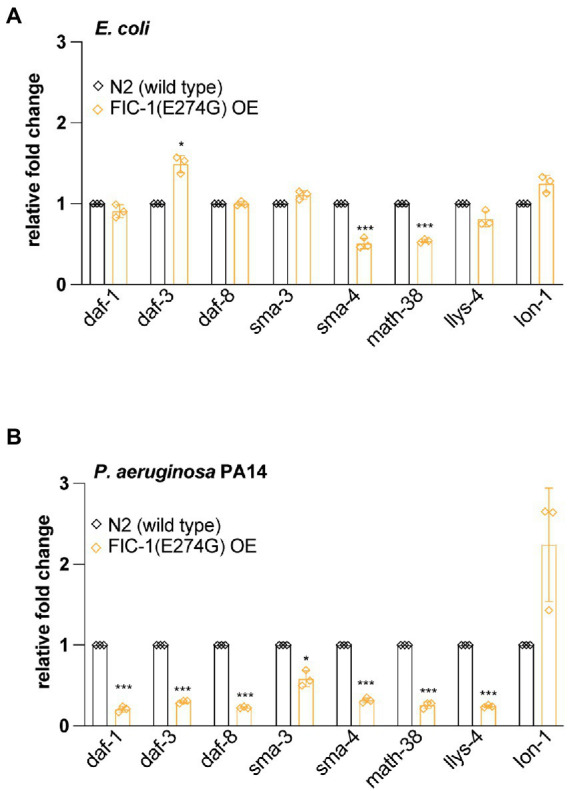
FIC-1(E274G)::HA OE alters expression of TGF-β signaling components. Uninduced and induced N2 wild type and FIC-1(E274G)::HA OE strains were kept on *E. coli* **(A)** and *P. aeruginosa* PA14 **(B)** plates and thereafter collected. Following RNA extraction, gene expression of indicated genes was determined by qPCR. Experiment was performed in triplicate with >200 animals per replicate/condtion. Error bars represent SD. Statistical significance was calculated using unpaired *t*-tests. ***p < 0.001, *p < 0.05, not significant (ns) *p* > 0.05.

To obtain a more comprehensive picture of how FIC-1(E274G)::HA OE affects TGF-β signaling, we collected control and FIC-1(E274G)::HA OE animals grown on OP50 and *P. aeruginosa* PA14 and extracted total RNA. We then determined expression levels of *daf-1*, *daf-3*, *daf-8* (required for DAF-7-dependent TGF-β signaling), *sma-3*, *sma-4* (required for DBL-1-dependent TGF-β signaling), *math-38*, *lys-4* [regulated by DAF-7-dependent TGF-β signaling (Hu et al., 2020)], and lon-1 [regulated by DBL-1-dependent TGF-β signaling (Maduzia et al., 2002)] by quantitative PCR (qPCR). We found that FIC-1(E274G)::HA OE in worms kept on *E. coli* led to a modest downregulation of *sma-4* and *math-38*, while *daf-3* was slightly upregulated (Figure 5A). Contrasting, FIC-1(E274G)::HA OE in worms exposed to *P. aeruginosa* PA14 promoted a significant down-regulation of all tested genes expect for lon-1 (Figure 5B), indicating that pathogen exposure drastically changed the impact of FIC-1(E274G)::HA OE on cellular signaling.

## Discussion

Fic-type AMPylases are critical regulators of eukaryotic signaling processes. In this study, we explored the physiological consequences of FIC-1(E274G)::HA OE in the nematode *C. elegans*. Overall, our phenotypic assessment showed that whole-body FIC-1(E274G)::HA OE has severe consequences on animal health, stress resilience, reproduction, innate immunity, and behavior.

Perhaps the most important finding of our work is the novel link between TGF-β signaling and FIC-1 activity. TGF-β signaling is a highly conserved program critically involved in essential cellular processes including cell growth and differentiation, and cell migration (Tzavlaki and Moustakas, 2020). *Caenorhabditis elegans* have five TGF-β family ligands: *dbl-1*, *daf-7*, *unc-129*, *tig-2*, and *tig-3*. DBL-1 and DAF-7 are both monomeric ligands binding to DAF-4/SMA-6 and DAF-4/DAF-1-containing type I/II receptors. The physiological roles of UNC-129, TIG-2, and TIG-3 remain largely unclear. Our results show that FIC-1(E274G)::HA OE prevents the production of DAF-7 in ASJ neurons in response to *P. aeruginosa PA14* exposure and the synthesis of DBL-1 in ASI and ASJ neuron in general. We also find that kd of HSP-1, the major cytosolic HSP70 family chaperone, is sufficient to phenocopy the loss of aversive behavior as observed in FIC-1(E274G)::HA OE animals. One possible explanation for these results is that FIC-1(E274G)::HA-mediated AMPylation of HSP-1 prevents HSP-1-assisted DAF-7 and/or DBL-1 folding. Despite considerable efforts, we did not find any indication of FIC-1(E274G)::HA-mediated DAF-7 and DBL-1 AMPylation (data not shown). However, we cannot exclude alternative HSP-1-independent mechanisms to be involved. Further work is required to decipher the mechanistic details for FIC-1-mediated regulation of TGF-β pathway.

Interestingly, we observed that in *nIs733* animals expressing FIC-1(E274G) at physiological levels, the ER-resident chaperone HSP-4, but not HSP-3, is required to elicit a protective avoidance response to *P. aeruginosa* PA14. This finding implies that the cellular function of HSP-3 and HSP-4 are unlikely to fully overlap, despite the high similarity and conservation of the two ER chaperones. This conclusion is consistent with previous studies showing that basal expression and stress inducibility of *hsp-3* and *hsp-4* are different (Shen et al., 2001; Zha et al., 2019). We also interpret these results as a strong indicator that physiological FIC-1 signaling, and not only FIC-1(E274G)::HA OE driven cellular processes are involved in the regulation of TGF-β signaling. The finding that HSP-1 is required to initiate an aversive response to *P. aeruginosa* PA14 in all tested genetic backgrounds suggests an AMPylation-independent role for this major chaperone in regulating worm behavior. The mechanistic details of this novel link remain to be determined.

Our work leaves us with several interesting questions: How does FIC-1(E274G)::HA OE result in behavioral avoidance defects in *daf-3* null backgrounds? Is there a role for DBL-1 in aversive behavior? And what are the mechanistic processes that connect FIC-1 activity to the regulation of TGF-β signaling? Based on our results, we propose a model in which FIC-1-mediated HSP-1, HSP-3, and HSP-4 modification influences DAF-7 and DBL-1 production, folding, and secretion. Our findings that HSP-1 kd phenocopies FIC-1(E274G) OE-associated phenotypes is in line with previous work showing that HSP-1 kd improved survival of amyloid β-expressing nematodes similar to endogenous level FIC-1(E274G) expression (Truttmann et al., 2018). We also showed previously that HSP-1 is directly modified by FIC-1(E274G) *in vitro* (Truttmann et al., 2016). While the increasing number of non-ER-associated FICD/FIC-1 targets strongly suggests that this enzyme is not strictly restricted to the ER lumen, the mechanistic basis for FICD/FIC-1-dependent protein AMPylation outside of the ER remains to be determined. We further propose that the FIC-1(E274G)::HA-dependent modification of HSP-3 and HSP-4 will directly affect UPR^ER^ signaling in *C. elegans* and, perhaps in parallel to cytosolic HSP-1 regulation, alter signal transduction through TGF-β signaling pathways. It’s also tempting to speculate that the exposure to pathogens, such as *P. aeruginosa* PA14, could change the activity of endogenous FIC-1 to provoke downregulation of the TGF-β pathway, as a protective mechanism against the accumulation of excessive misfolded protein loads.

In conclusion, our work proposes the involvement of FIC-1-mediated protein AMPylation in the regulation of TGF-β signaling in the nematode *C. elegans*. We also describe for the first time a direct behavioral consequence of altered FIC-1-mediated protein modification. Further studies focusing on the links between physiological FIC-1 function and TGF-β pathway regulation should provide additional insights into the role of FIC-1 in proteostasis and cellular signaling.

## Materials and methods

### *Caenorhabditis elegans* strains and growth conditions

All strains were grown on nematode growth medium (NGM) plates seeded with *E. coli* OP50 at 20°C unless strains were considered temperature-sensitive, in which case they were grown at 15°C. Hermaphrodites were used in the study. The strains used in this manuscript are described in Supplementary Table S1.

### Worm synchronization

For all behavioral assays, a synchronized population of worms was used. Briefly, worms were washed off NGM plates, collected by sedimentation, washed twice with water and once with M9 [3 g KH_2_PO_4_, 6 g Na_2_HPO_4_, 5 g NaCl in 950 ml MilliQ]. Worms were then treated with hypochlorite bleaching buffer [56.6 ml of distilled water with 14.4 ml of 5 N NaOH and 6.6 ml of 8.25%NaHOCl] and incubated for 7 m at 20°C with agitation (1,200 rpm). Immediately afterwards, samples were centrifuged for 30 s at 100 g followed by 2 washes with water. Embryos were then transferred to OP50-seeded NGM plates until they reached day 1 adulthood. Day 1 adults were transferred to either *Pseudomonas aeruginosa* PA14*, E. coli* OP50, or RNAi-plates.

### *Pseudomonas aeruginosa* avoidance and survival assays

Experiments were performed as described in (Truttmann et al., 2016). Briefly, *Pseudomonas aeruginosa* isolate 14 (PA14) was cultured overnight in 5 ml LB broth at 37°C as described before. For avoidance assays, Slow-killing assay (SKA) plates were seeded with 7 μl of *P. aeruginosa* PA14 and maintained at 37°C overnight, and 20°C for an additional 24 h. Animals were placed in the center lawn of *P. aeruginosa* P14 at day 1 or day 4 of adulthood and kept at 25°C for 20 h after which avoidance behavior was scored by quantifying the number of worms remaining in the *P. aeruginosa* PA14 lawn compared to the number of worms outside the *P. aeruginosa* PA14 lawn. For *P. aeruginosa* PA14 survival assays, *C. elegans* survival was scored daily. Animals were considered dead if repetitive poking (10×) did not result in any visible body movement. Dead animals were immediately removed from assay plates. PA14 experiments were performed at 25°C, which is sufficient to induce expression of FIC-1(E274G)::HA from allele nIs774 without additional heat induction. For Tunicamycin tests, worms were grown for 72 h on NGM agar plates containing 0.25 mg/ml Tunicamycin and transferred to *P. aeruginosa* PA14 plates on day 1 of adulthood.

### *Caenorhabditis elegans* microscopy

To assess expression levels of fluorescent proteins in individual neurons, animals were mounted in M9 with Tetramisole (1 mM) onto slides with 1% agarose pads. Images were acquired on a Keyence BZ-x700 fully automated fluorescence microscope with a 40× Plan Apo Lambda objective (air, NA = 0.95). Maximum fluorescence intensity was quantified using Image J FIJI software (Schindelin et al., 2012).

### RNA interference

Knock-down of chaperones (*hsp-1, hsp-3, and hsp-4*) was induced by RNAi feeding. Knockdown efficiency and target specificity for each RNAi was validated by qPCR (data not shown). *E. coli* HT115 expressing the appropriate small interfering RNA precursors were grown in LB containing 100 μg/ml carbenicillin at 37°C overnight. The following day, *E. coli* HT115 cultures were concentrated and induced with 5 mM IPTG. NGM growth media was supplemented with 100 μg/ml carbenicillin, 1 mM isopropyl β-D-1-thiogalactopyranoside (IPTG) and plates were seeded with the appropriate RNAi bacteria. For combinatorial experiments, equal amounts of each HT115 *E. coli* strain expressing the appropriate small interfering RNA precursors were seeded as a single lawn on the same NGM plate. Nematodes were transferred to RNAi plates on day 1 of adulthood and incubated at 20°C for 1–3 days, depending on the experiment. *E. coli* HT115 expressing small interfering RNA precursors specific for *gfp* and *pos-1* were used as controls.

### Dauer development assays

For dauer assays, embryos were collected from hypochlorite-treated gravid adults. L1 larvae were incubated at 27°C or 20°C for 72 h and subsequently scored. Quantification of animals in dauer-stage was done *via* identification of the unique physical phenotype of dauer animals.

### Growth measurements

At indicated timepoints, control and RNAi-treated animals were transferred to non-spotted NMG plates to remove any OP50 bacteria. Animals were then immobilized with Tetramisole (1 mM) on a 1% agarose pad and imaged using a MZ10 Leica microscope at 3.2×. Images were analyzed using ImageJ FIJI software to quantify major body axis length.

### FIC-1(E274G)::HA transgene induction

To induce FIC-1(E274G) expression, animals were incubated at 37°C for 30 min followed by 30 min at 20°C for recovery.

### Image quantification

We used Fiji (Schindelin et al., 2012) to quantify GFP signal intensity in ASI and ASJ neurons. For this, we first placed an oval outline around each neuron and only quantified the signal within this area. The same oval outline was used for all images within the same replicate.

### Quantitative PCR

qPCR was performed as previously described (Truttmann et al., 2018). In brief, total RNA was isolated, reverse transcribed using the iScript reverse transcription Supermix for RT-PCR (biorad) and analyzed on a QuantStudio 6 flex real time PCR system. Supplementary Table S2 lists all primers used in RT-qPCR tests.

### Experiment statistics

Statistical analyses were performed in Graphpad Prism (GraphPad Software). The individual statistical tests utilized in each experiment as well as number of worms per group are indicated in the respective figures and figure legends.

## Data availability statement

The raw data supporting the conclusions of this article will be made available by the authors, without undue reservation.

## Author contributions

MCT supervised the project. MCT, MHL, and ZM planned and designed the experiments. MHL, MC, ZM, and MCT performed all experiments. MCT and MHL wrote the manuscript. All authors contributed to the article and approved the submitted version.

## Funding

MH-L received support from a Rackham fellowship, NIA Training grant AG000114, and NIH F31 grant 1F31DC020397-01. MT is supported by the Alzheimer’s foundation, National Ataxia foundation, Ruth K Broad foundation and NIH grant 5R35GM142561-02.

## Conflict of interest

The authors declare that the research was conducted in the absence of any commercial or financial relationships that could be construed as a potential conflict of interest.

## Publisher’s note

All claims expressed in this article are solely those of the authors and do not necessarily represent those of their affiliated organizations, or those of the publisher, the editors and the reviewers. Any product that may be evaluated in this article, or claim that may be made by its manufacturer, is not guaranteed or endorsed by the publisher.
