# Supplementary figures and images for: The AMPylase FIC-1 modulates TGF-β signaling in Caenorhabditis elegans
Source: Front Mol Neurosci. 2022 Nov 24;15:912734. doi: 10.3389/fnmol.2022.912734 (PMC9730714; doi:10.3389/fnmol.2022.912734)

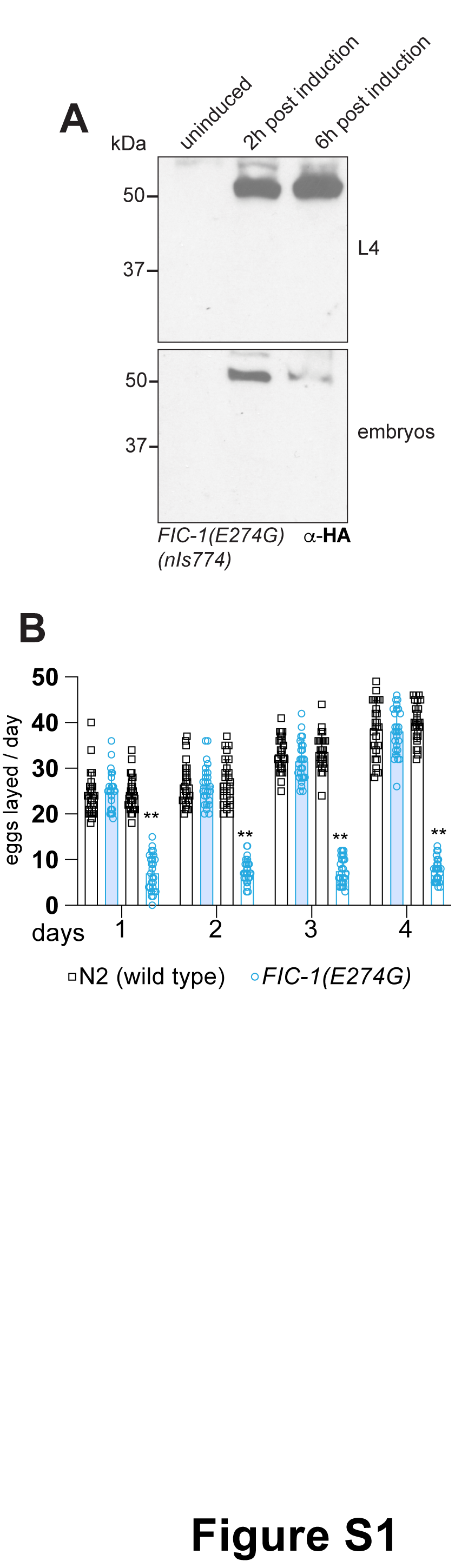

Supplement: Supplementary file 3 [file Image_1.tif]

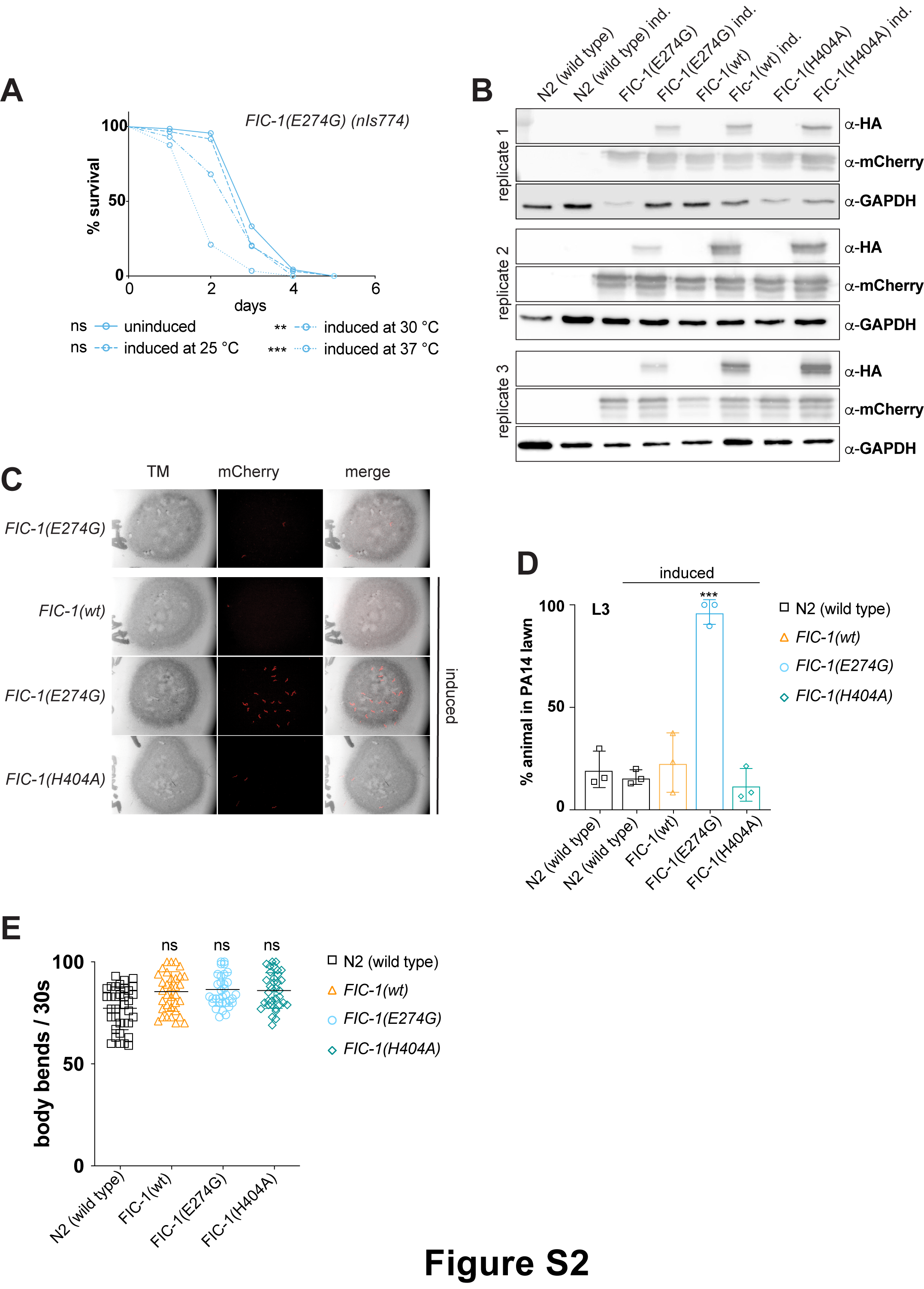

Supplement: Supplementary file 4 [file Image_2.tif]

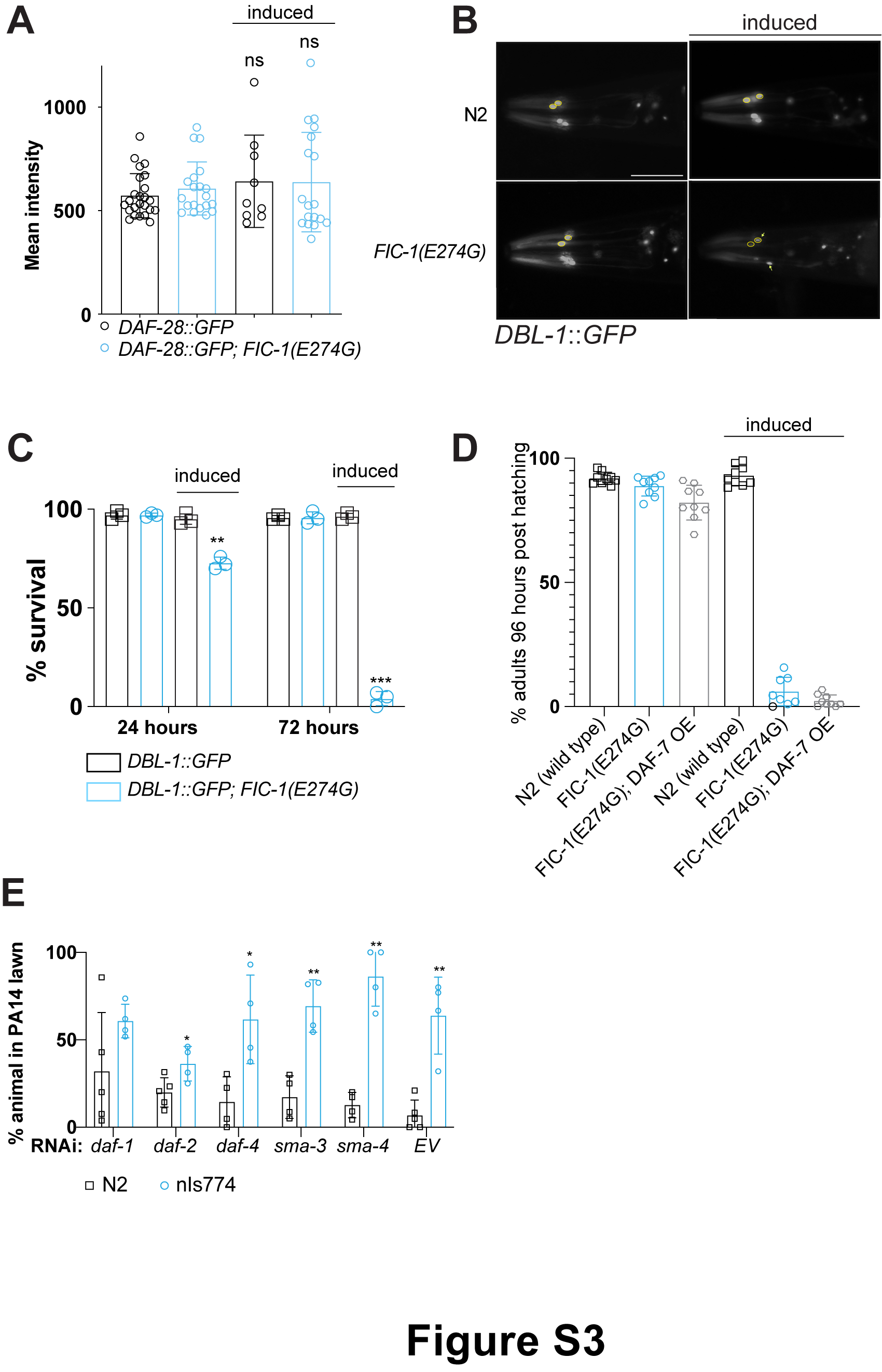

Supplement: Supplementary file 5 [file Image_3.tif]

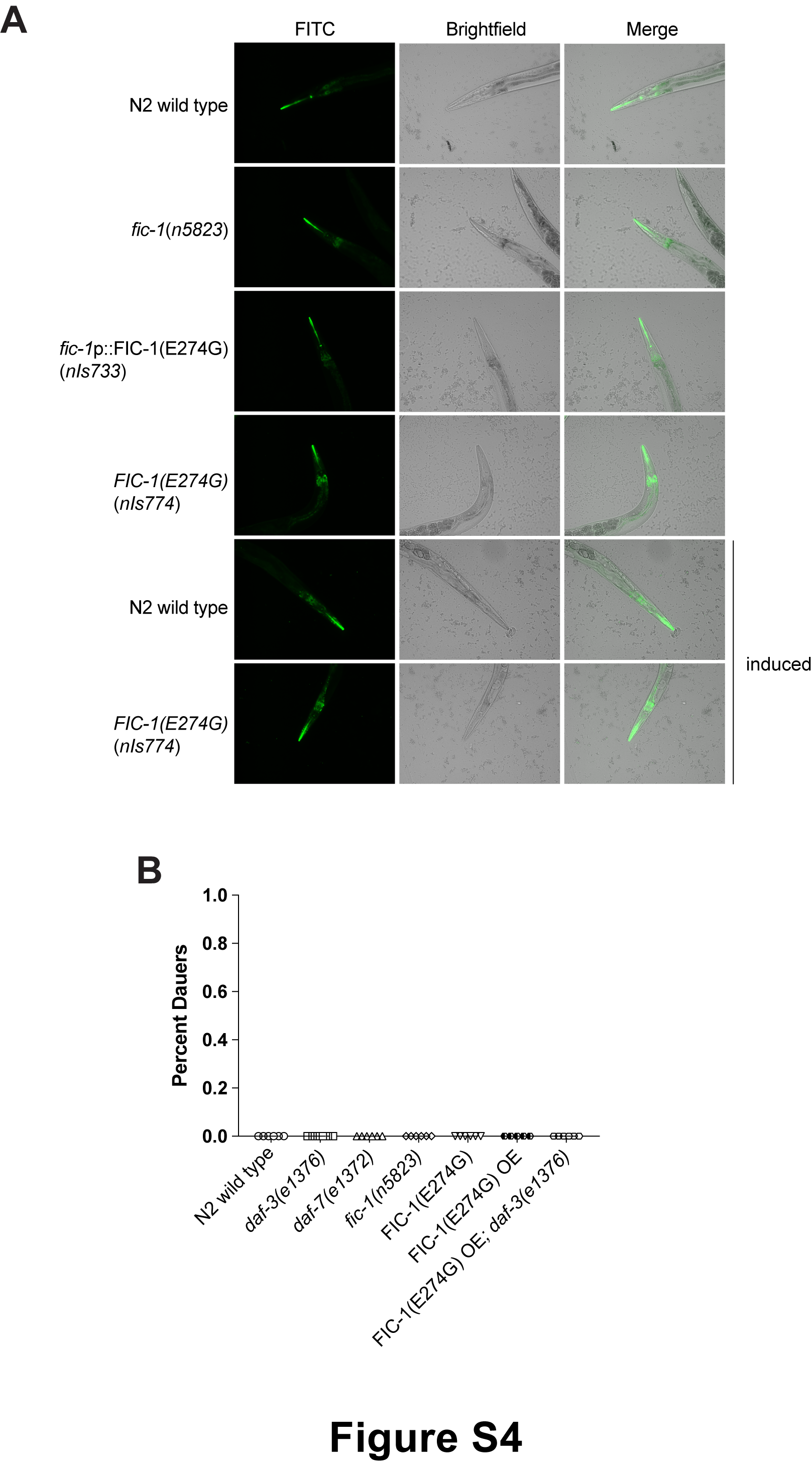

Supplement: Supplementary file 6 [file Image_4.tif]
